# Antibiotic resistance and virulence patterns of pathogenic *Escherichia coli* strains associated with acute gastroenteritis among children in Qatar

**DOI:** 10.1186/s12866-020-01732-8

**Published:** 2020-03-06

**Authors:** Nahla O. Eltai, Asmaa A. Al Thani, Sara H. Al Hadidi, Khalid Al Ansari, Hadi M. Yassine

**Affiliations:** 1 Biomedical Research Center, Qatar University, P.O. Box 2713, Doha, Qatar; 2 College of Health Sciences, Qatar University, QU Health, Doha, Qatar; 3 Pediatrics Emergency Center, Hamad Medical Corporation, Doha, Qatar; 4 Emergency Medicine Department, Sidra Medicine, Doha, Qatar

**Keywords:** Antibiotic resistance, DEC, EPEC, EAEC, ESBL, MDR, Virulence

## Abstract

**Background:**

The treatment of *Enterobacteriaceae* family including diarrheagenic *E. coli* (DEC) has been increasingly complicated due to the emergence of resistant strains. Here we report on the phenotypic resistance profiles and ESBL genotype and virulence profiles of Enteroaggregative *E. coli* (EAEC) and Enteropathogenic *E. coli* (EPEC) isolated from children hospitalized with acute gastroenteritis in Qatar (AGE).

**Results:**

*E. coli* were isolated and characterized from 76 diarrheagenic stool positive samples, collected from hospitalized children less than 10 years old. Isolates were tested for antibiotic susceptibility against eighteen clinically relevant antibiotics using E-test method. Conventional PCR was performed to detect genes encoding ESBL and virulence factors. Chi-square test was performed to compare the individual antibiotic resistance between EPEC and EAEC.

A significant percentage (73.7%) of isolates were resistant to at least one antibiotic. Overall, high resistance (70%) was reported to the first-line antibiotics such as ampicillin, tetracycline (46.4%), and sulfamethoxazole-trimethoprim (42.9%). Further, 39.5% of the isolates were multidrug resistant (MDR), with 22.4% being ESBL producers. On the other hand, all isolates were susceptible to carbapenem, fosfomycin, amikacin and colistin. The incidences of resistance to the 18 antibiotics between EPEC and EAEC were not significantly different by Pearson chi -square test (*P* > 0.05). Genetic analysis revealed that 88.23% of ESBL production was *bla*_CTX-M-G1_ (*bla*_CTX-M-15_, *bla*_CTX-M-3_) - encoded. Several different combinations of virulence markers were observed, however, there was no specific trend among the isolates apart from absence of the bundle-forming pilus *(bfpA*) gene, which encodes the type IV fimbriae in EPEC adherence factor (EAF) plasmid (pEAF), among all EPEC (atypical). 15% of the EAEC strains were positive for a combination of *astA*, *aap* & *capU*, while 10% were positive for three different combinations. The *aap*, *aatA*, *capU* and *aggR* virulence genes showed the highest frequency of 65, 60, 55 and 55% respectively. Others genes, *east*, *astA*, and *aai*, showed frequencies of 35, 30 and 20% respectively.

**Conclusions:**

Atypical EPEC and EAEC were the primary etiological agents of diarrhea in children among DEC pathotypes. Our results indicated high rate of antimicrobial resistance pattern of DEC strains, which necessities the development of regulatory programs and reporting systems of antimicrobial resistance in DEC and other AGE-associated bacteria to insure effective control of diarrheal diseases. Results from this study demand a further research on identifying the phenotypic and genotypic profiles of more DEC pathotypes in various clinical samples.

## Background

Different pathotypes of diarrheagenic *Escherichia coli* (DEC) are the main cause of pediatric diarrhea worldwide, particularly in developing countries [1, 2] and travelers to those countries. DEC strains have been classified into five main types based on their specific virulence factors, clinical manifestations of the disease, epidemiology and phylogenetic profile. These bacteria are Enteroaggregative *E. coli* (EAEC), Enteropathogenic *E. coli* (EPEC), Enterotoxigenic *E. coli* (ETEC), Enteroinvasive *E. coli* (EIEC), and Enterohemorrhagic *E. coli* (EHEC) [3]. The progressive increase of antibiotic resistance (AR) continues to pose a great threat to public health in both developed and developing countries [4, 5]. The treatment of *Enterobacteriaceae* family, including *E. coli*, has been increasingly complicated by the emergence of resistant strains to most first-line antimicrobial agents [6, 7]. Many patients with gastroenteritis are empirically treated with antibiotics, which could be ineffective in many cases such as ampicillin, and ciprofloxacin in adults. This misuse of antibiotics in treating diarrhea, especially in the developing world where the rate of diarrheal diseases is the highest and the use of antimicrobial agents is often indiscriminate, could lead to increased AR [8]. A distressing increase in multi-drug resistant enterobacteriaceae, particularly to third-generation cephalosporins and colistin (last resort antibiotic used to treat carbapenem-resistant enterobacteriaceae), has been reported in different regions [9–12].

Information about AR among DEC is important in selecting the appropriate therapy. Little is known about AR profile of DEC isolated from diseased children in the Middle East and North Africa region (MENA) [13]. In a study among pediatric patients who were admitted to Jeddah hospital, the prevalence of enteropathogenic *E. coli* was 3.8% and enterohaemorrhagic was 1.9% [14]. In another study from the United Arab Emirate, the prevalence of ESBL among EAEC isolated from children presented with diarrhea was 11.3%. The objectives of this study are to: (1) determine the prevalence of EAEC and EPEC, the most prevalent, among children suffering from acute gastroenteritis in Qatar; (2) determine phenotypically and genotypically the AR profiles; and (3) determine the prevalence of virulence genes in these DEC.

## Methods

### Clinical isolates

A total of 175 fecal samples were collected between August 2017 and January 2108 from children (0–10 years of age) of different nationalities, hospitalized with AGE associated with diarrhea, vomiting and fever, at the Pediatric Emergency Center (PEC)-Hamad Medical Corporation (HMC). All samples were collected with informed consent signed by the parents/legal guardians under IRB approval # 16173/19 from HMC and Qatar University approval number MRC-16173/16 and QU-IRB605-E/16, respectively. For each individual, demographic data such as age, nationality, and gender were collected. Samples were initially screened with Film Array Gastrointestinal (GI) Panel kit (BIOFIRE®, Cambridge, USA) for viral, bacterial and parasitic agents associated with AGE. Leftover stool samples (~ 0.5 g) were individually diluted into 4 ml of PBS each to get the stool suspension. Ten percent glycerol was added to each tube before storing at − 80 ^o^ C for downstream applications. In total, 76 fecal samples were utilized in this study, 56 of which were EPEC and 20 were EAEC as detected with Film Array GI kit.

### Bacterial culture

To isolate *E. coli*, 20 μl of stool suspension was inoculated and streaked directly onto a selective medium CHROMagar™ *E. coli* plates (Difco, Becton Dickenson, Sparks, MD) using sterile cotton-tipped swabs, and then incubated at 37 °C for 18–24 h. A typical single *E. coli* colony (green color with smooth surface) was randomly selected and subsequently streaked onto MacConkey agar (Difco, BD) plates and incubated at 37 °C for 18–24 h. Lactose fermenter pink dry colonies were selected and streaked onto fresh blood agar plates and incubated again at 37 °C for 18–24 h. Colonies from the blood agar were tested for conversion of tryptophan into indole using Indole spot test (Remel, Thermoscientific, KS, USA). *E. coli* colonies were further confirmed biochemically by BIOMIC V^3^ (Giles Scientific, USA). The confirmed *E. coli* isolates were transferred to Cryovial tubes (TechnicalService, Lancashire, U.K.) and stored at − 80 °C until further analysis.

### DNA extraction and polymerase chain reaction (PCR)

PCR was used to differentiate DEC into EPEC and EAEC based on the presence of virulence genes. First, DNA was extracted from bacterial cultures using QIAamp® UCP pathogen mini Kit (Qiagen, Germany) following manufacturer’s instructions and then used to run a combination of uni- and multiplex PCR assays targeting 12 genes using previously published primers (Table 1). Uniplex PCR was performed to detect *eae*, *tir, bfpA* and *capU* virulence genes. Conditions for reactions were as follows: PCR mixture was made in volume of 20 μl containing 0.5 μM of each pair of primers, 30 ng DNA, 10 μl of Hot star *Taq* plus master mix (Qiagene, Germany), 1x of Corolload load concentrate and DPEC H2O up to 20 μl. The reactions were amplified in Biometra TAdvanced thermocycler (analyticjena, Jena, Germany). Conditions for amplification were carried out as described in previous studies [15–19]. The Multiplex PCR (MPCR) was performed to detect *aap* and *aatA* genes (MPCR1), *aai* and *astA* genes (MPCR2), *aggR* and *east* (MPCR3) and MPCR4 to detect *sxt*1 and *sxt*2. MPCR1 was performed in a final volume of 30 μl, containing 0.5 μM of each primer, 30 ng DNA, 15 μl master mix (Hot star *Taq* plus master mix (Qiagen, Germany), 1x Corolload load concentrate, and DPEC H2O up to 30 μl. MPCR2 was performed in a final volume of 25 μl containing 0.4 μM of each primer, 30 ng DNA, 12.5 μl master mix (Hot star *Taq* plus master mix (Qiagene, Germany), 1x Corolload load concentrate, and DPEC H2O up to 25 μl. MPCR3 and MPCR4 were performed in a final volume of 30 μl, containing 0.5 μM of each primer, 30 ng DNA, 15 μl master mix (Hot star *Taq* plus master mix (Qiagen, Germany), 1x Corolload concentrate, and DPEC H2O up to 30 μl. Amplified products were subjected to electrophoresis in 1.2% agarose (Agarose- LE, Ambion®, USA), stained with 0.2 mg/ml ethidium bromide (Promega, Madison, USA), and visualized using iBright CL1000 imaging system (invitrogen, US).

**Table 1:** Primers used to amplify selected pathogenic *E. coli* virulence genes

Organism	Primer sequence (5′ → 3′)	Target gene	Size (bp)	Reference
Enteropathogenic *E.coli* (EPEC)	GACCCGGCACAAGCATAAGC CCACCTGCAGCAACAAGAGG	*eae*	384	[15]
EPEC	CAGCCTTCGTTCAGATCCTA GTAGCCAGCCCCCGATGA	*tir*	400	[16]
EPEC	AATGGTGCTTGCGCTTCGTGC GCCGCTTTATCCAACCTGGTA	*bfpA*	326	[15]
Enteroaggregative *E.coli* (EAEC)	CTA ATT GTA CAA TCG ATG TA AGA GTC CAT CTC TTT GAT AAG	*aggR*	457	[17]
EAEC	CTT GGG TAT CAG CCT GAA TG AAC CCA TTC GGT TAG AGC AC	*aap*	310	[17]
EAEC	CTG GCG AAA GAC TGT ATC AT CAATGT ATA GAA ATC CGC TGT T	*aatA*	629	[17]
EAEC	ATGAATATACTATTTACGGAATC CTACAGGCACAGAAAATGCCGATG	*capU*	776	[16]
EAEC	CTC TTA GCA GGG AGT TTG TC GCT TTG TTT ACC GAC TGA AC	*aaiA*	430	[18]
EAEC	CCA TCA ACA CAG TAT ATC CGA GGT CGC GAG TGA CGG CTT TGT	*astA*	111	[18]
EAEC	CACAGTATATCCGAAGGC CGAGTGACGGCTTTGTAG	*east*	97	[19]
* EPEC/EHEC	ATAAATCGCCTATCGTTGACTAC AGAACGCCCACTGAGATCATC	*SXT1*	180	[15]
* EPEC/EHEC	GGCACTGTCTGAAACTGCTCC TCGCCAGTTATCTGACATTCTG	*SXT2*	225	[15]

*The Enterohemorrhagic *E. coli* (EHEC) pathotype can be identified by the presence of the *eae* gene along with *stx1* gene, *stx2* gene, or both [20]. Therefore, to differentiate between EHEC and EPEC we screen for the presence of *stx1* gene, *stx2* gene

### Antimicrobial susceptibility testing

Antimicrobial susceptibility test was performed using standard E test strips (E-test strip Diagnostic Liofilchem®, Italy) technique in accordance with the recommendations of CLSI, 2017 [21]. Zone of inhibition was examined to determine the minimum inhibitory concentration [22] values that were interpreted according to the CLSI guidelines [21]. ATCC *E. coli* strains number 25922 and 35,218 were used as susceptible and β-lactamase producing control strains9, respectively. The 18 antibiotics used to screen the antibiotic susceptibility of EPEC and EAEC are colistin, piperacillin/tazobactam, fosfomycin, ciprofloxacin, nitrofurantoin, amikacin, ampicillin, cephalothin, cefuroxime, ceftriaxone, cefepime, tigecycline, amoxicillin /clavulanic acid, ertapenem, meropenem, trimethoprim/sulfamethoxazole, tetracycline, and chloramphenicol. The 18 clinically relevant antibiotics used to screen the antibiotic susceptibility of *E. coli* are summarized in Supplementary 1.

### Phenotype confirmation and molecular genotyping of extended-spectrum β-lactamase (ESBL) genes

In recent years, there are major concern about antibiotic resistant genes encoding ESBLs. The ESBL confer resistance to penicillins and cephalosporins 1st, 2nd & 3rd generations and are non-inhibited by inhibitors such as clavulanic acid and tazobactam. ESBL producing strains often exhibit multidrug resistance, including resistance to aminoglycosides and fluoroquinolones, thus limiting the therapeutic options. β- Lactam antibiotics are the most widely used for all systemic infections. For these reasons, this study has concerned to identify the occurrence of ESBL among DEC and detect the genes responsible for ESBL production.

Isolates that were tested positive for third generation cephalosporin were consequently confirmed by Double Disc Synergy Test, (DDST) as previously described [21, 23]. Molecular genotyping of isolates exhibiting ESBL was performed by characterization of the presence/absence of 10 genes namely, *bla*_CTX-M-G (1, 2, 8, 9, &25)_, *bla*_TEM_ and *bla*_SHV_ as described by [24]. Furthermore, in this study we targeted the presence of *bla*_CTX-M − 14_ and stratified the CTX-M-G1 to specify CTX-M-3 or CTX-M-15 type’s resistant genes. We identify them with PCR product size of 335, 479, and 996 bp respectively, by application of uniplex and multiplex PCR. Briefly, PCR reactions were performed in a total volume of 20 μl containing 0.5 pmol of each two pairs of previously published primers namely, F-5’CACACGTGGAATTTAGGGACT3’ and R-5′ GCCGTCTAAGGCGATAAACA3’ [25] for *bla*_CTX-M-15_; F-5’AATCACTGCGCCAGTTCACGCT3’ and R- 5’GAACGTTTCGTCTCCCAGCTGT3’ for *bla*_CTX-M-3_ [26]; F-5’TACCGCAGATAATACGCAGGTG3’ and R-5′ CAGCGTAGGTTCAGTGCGATCC 3′ for *bla*_CTX-M-G-14_ [26]. 10 μl of HotStar *Taq* plus master mix (Qiagen, Germany), 2 μl of 1 x Corolload concentrate, 2 μl of DNA samples and DPEC H_2_O up to 20 μl. The reactions were amplified in Biometra TAdvanced thermocycler (Analyticjena, Jena, Germany). NCTC *E. coli* strains 13,461, 13,462, and 13,463; *Enterobacter cloacae* 13,464, *E. coli* 13,353 and *Klebsiella pneumoniae* 13,465*, E. coli* ATCC 35218 and *E. coli* NCTC 13368 were used as positive controls in PCR assays for CTX-M G1, CTX-M G2, CTX-M G8, CTX-M G9, CTX-M G15, CTX-M G25 *bla*_TEM_ and *bla*_SHV_, respectively. Amplified products were subjected to electrophoresis in 1.2% agarose (Agarose- LE, Ambion®, USA), stained with 0.2 mg/ml ethidium bromide (Promega, Madison, USA), and visualized using iBright CL1000 imaging system (invitrogen, US).

### Sequencing and sequence analysis

Amplicons products that obtained from PCR reactions targeting virulence genes were purified by ExoSAP-IT (GE Healthcare life science, Chicago, USA) according to the manufacturer instructions, and then subjected to sequencing reactions using specific forward and reverse primers for each gene (Table 1) with Big DyeTerminator Reaction Mix (Applied Biosystems, USA). The reaction products were purified using Big Dye XTerminator purification Kit (Applied Biosystems) per manufacturer instructions and run on ABI 3500 XL sequencer (Fisher scientific, USA). The sequencing of the virulence gene amplicons were confirmed using on line NCBI blast tool.

### Data analysis

Data were introduced into Microsoft Excel 2010 (Microsoft Corporation, New York, USA) to generate figures and run initial analysis and further statistical analysis was performed using SPSS statistics 25 (Statistical Package for the Social Science; SPSS Inc., Chicago, IL, USA). To compare the individual antibiotic resistance between EPEC and EAEC, chi-square test was calculated using Pearson probability value (*P* value). *P*-value less than 0.05 was considered statistically significant.

## Results

### Demography of the study population

A total of 175 collected stool samples were screened for DEC. Only those intreperpreted as positive for EPEC and EAEC were included in the study and characterized in details.

The demographic profile of the studied population is summarized in Table 2. About 55% of samples were collected from males compared to 44.7% from females (0–10 years of age), with female to male ratio of 1:1.2. DEC were more prevalent among Qataris (43.4%) compared to other nationalities: Pakistani (10.5%); Egyptians (9.2); Syrian (9.2%); Indians (6.6%); Iranian and Sudanese (3.9%); Algerian, Yemeni and Filipino (2.6%); and American and Moroccan (1.3%). Most of the DEC detected during this study were among children less than 2 years of age (59.2%), compared to those of the age between 2 and 5 years (23.6%) and 6–10 years (17%).

**Table 2:** Demographic profile of the study population (76) with DEC in the State of Qatar

Age group (years)	Total number/percentage		Nationality (Total number/percentage)	
	Male	Female	Qatari	Non Qatari (n* = 12)
< 2	26 (34.2%)	19 (25%)	17 (22.4%)	28 (36.8%)
2–5	11 (14.5%)	7 (9.2%)	10 (13.2%)	8 (10.55)
6–10	5 (6.6%)	8 (10.5%)	6 (7.9%)	7 (9.2%)

*Represent the number of nationalities tested

### Pathotyping of DEC in stool samples from children with AGE

According to the film array testing our 175 diarrheagenic stools samples from AGE children were classified as EPEC (56/175, 32%), the most predominant pathogen followed by EAEC (20/175, 11.43%), then EIEC (8/175, 4.6%), ETEC (1/175, 0.6%) and the rest other causes. Only EPEC and EAEC were further tested for their AMR profile due to their significant numbers.

### Phenotypic resistance profile

**Fig. 1:**
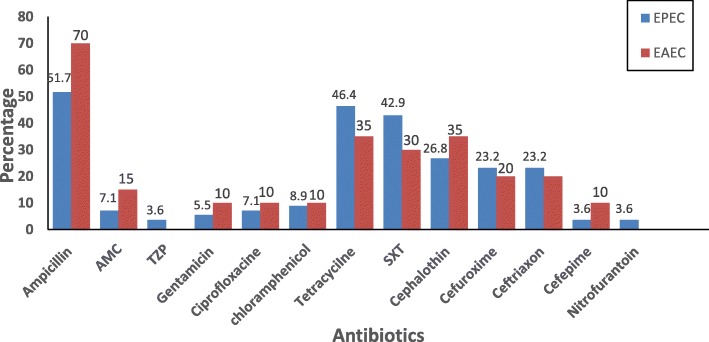
Comparison of phenotypic antimicrobial resistance profile of EPEC (56) and EAEC (20) isolated from children (age 0 to 10 years) suffering from AGE. The figure depicts the percentage of isolates with resistance to 14 of the 18 tested antibiotics. AMC: Amoxicillin/Clavulanic acid; TZP: Piperacillin/ Tazobactam; SXT: Trimethoprim/Sulfamethoxazole; *P* > 0.05 for the comparison between EPEC and EAEC against all antibiotics

The percentage of the antibiotics resistance profile against 56 EPEC and 20 EAEC is depicted in Fig. 1. In general, EPEC and EAEC isolates were respectively showing high resistance to ampicillin (51.7, 70%), followed by tetracycline (46.4, 35%), trimethoprim/ sulfamethoxazole, (42.9, 30%), and cephalosporins: cephalothin (26.8, 35%), cefuroxime (23.3, 20%) and ceftriaxone (23.3, 20%). Relatively, less resistance was recorded against amoxicillin/clavulanic acid (7.1, 15%), gentamicin (5.5, 10%), cefepime (3.6, 10%) ciprofloxacin (7.1, 10%) and chloramphenicol (8.9, 10%). EPEC isolates exhibited 3 and 3.6% antibiotic resistance against piperacillin/tazobactam and nitrofurantoin, respectively whereas EAEC isolates were entirely susceptible. All EPEC and EAEC isolates were susceptible to meropenem, ertapenem, fosfomycin, colistin and amikacin. Thirteen isolates (23.2%) of EPEC as well as 4 isolates of EAEC (20%) were ESBL producers. Further 39.3% (22), and 40% (8) of EPEC and EAEC respectively (Table 3), were multidrug resistant (MDR): these are defined as acquired non-susceptibility to at least one agent in three or more antimicrobial categories [27]. The incidences of resistance to the 18 antibiotics between EPEC and EAEC were not significantly different by Pearson chi -square test (*P* > 0.05).

**Table 3:** Phenotypic resistant pattern of EPEC (*n* = 56) and EAEC (*n* = 20) isolates from children with AGE

Resistant phenotype	Frequency		Percentage	
	EPEC	EAEC	EPEC	EAEC
No resistance	16	4	28.6	20
*ampicillin, tetracycline, sxt	6	1	10.7	5
**#**ampicillin, cephalothin, cefuroxime, ceftriaxone	5	0	8.9	0
tetracycline	4	0	7.1	0
*chloramphenicol,tetracycline, sxt	2	0	3.6	0
*#ampicillin, sxt, cephalothin, cefuroxime, ceftriaxone	2	0	3.6	0
sxt	2	1	3.6	5
piperacillin	1	0	1.8	0
nitrofurantoin	1	0	1.8	0
ampicillin, amc, cephalothin	1	0	1.8	0
*ampicillin, amc, tetracycline, sxt	1	0	1.8	0
*ampicillin, gentamicin, ciprofloxacin, chloramphenicol, tetracycline, sxt	1	0	1.8	0
*ampicillin, chloramphenicol, tetracycline, cephalothin	1	0	1.8	0
*ampicillin, ciprofloxacin,tetracycline, sxt	1	0	1.8	0
tetracycline, sxt	1	0	1.8	0
*#ampicillin, tetracycline, sxt, cephalothin, cefuroxime,, ceftriaxone, cefepime	1	0	1.8	0
*ampicillin, chloramphenicol, tetracycline, sxt	1	0	1.8	0
*#ampicillin, chloramphenicol, tetracycline, sxt, cephalothin, cefuroxime, ceftriaxone	1	1	1.8	5
ampicillin, tetracycline	1	0	1.8	0
*#ampicillin, gentamicin, tetracycline, sxt, cephalothin, cefuroxime, ceftriaxone	1	0	1.8	0
*#ampicillin, tetracycline, sxt, cephalothin, cefuroxime, ceftriaxone	1	0	1.8	0
*#ampicillin, amc, tzp, ciprofloxacin, tetracycline, cephalothin, cefuroxime, ceftriaxone, cefepime	1	0	1.8	0
*#ampicillin, ciprofloxacin, tetracycline, sxt, cephalothin, cefuroxime, ceftriaxone	1	0	1.8	0
*ampicillin, amc, sxt	1	0	1.8	0
sxt, nitrofurantoin	1	0	1.8	0
ampicillin, tetracycline	1	1	1.8	5
ampicillin	0	4	0	20
*#ampicillin, amc, tetracycline, cephalothin, cefuroxime, ceftriaxone, cefpime	0	2	0	10
ampicillin, cephalothin	0	1	0	5
ampicillin, sxt	0	1	0	5
*ampicillin, chloramphenicol, tetracycline	0	1	0	5
*#ampicillin, ciprofloxacin, sxt, cephalothin, cefuroxime, ceftriaxone	0	1	0	5
*ampicillin, gentamicin, amc, cephalothin	0	1	0	5
*ampicillin, gentamicin, ciprofloxacin, cephalothin	0	1	0	5

*amc* amoxicillin/clavulanic acid, *sxt* trimethoprim/sulfamethoxazole, *MDR* multidrug resistant, *esbl* extended spectrum β- lactamase producer

_*_: MDR (*n* = 22 for EPEC and *n* = 8 for EAEC)

#: ESBL (*n* = 13 for EPEC and *n* = 4 for EAEC)

### Genotypic resistance profile

**Fig. 2:**
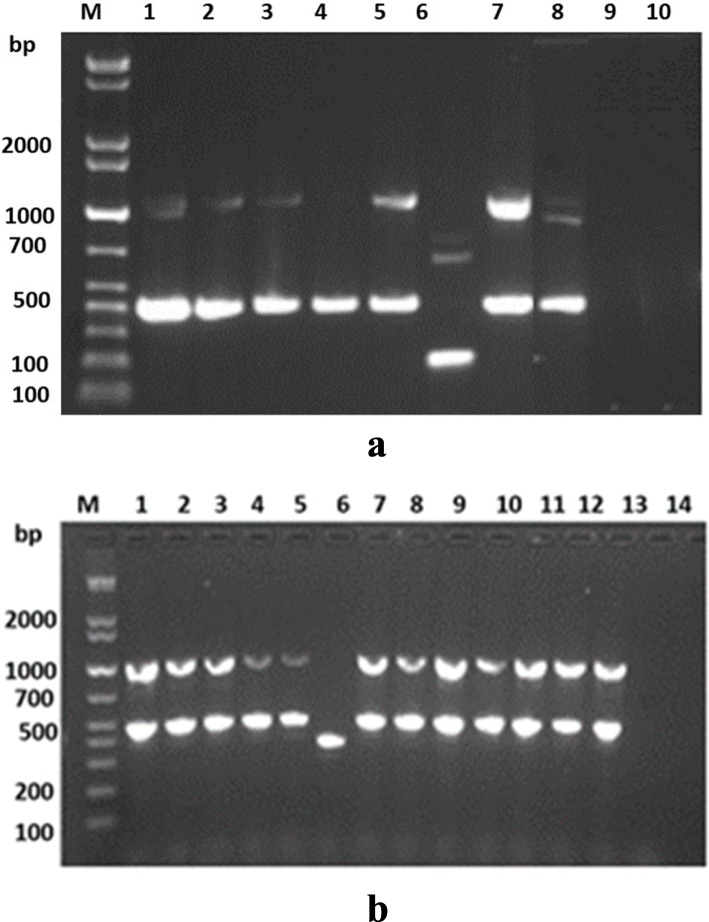
Detection of antibiotic resistance genes among 17 ESBL Enterobacteriaceae pathogens isolated from children with AGE. **a**: ^*bla*^SHV, ^*bla*^TEM and ^*bla*^CTX-M-G (1, 2, 8 &9). **b**: ^*bla*^CTX-M-G (3, 14 &15). Representative samples are shown. Multiplex PCR was performed for detection of CTX-M groups with exception of ^*bla*^CTXM-G15, while monoplex PCR was used for detection of TEM and SHV. The amplification products of each isolate were run on the same lane for detection of ^bla^ genes. **a** Lane 1: ^*bla*^CTXM-G1, ^*bla*^TEM, ^*bla*^SHV; Lane 2: ^*bla*^CTXM-G1, ^*bla*^TEM; Lane 3: ^*bla*^CTXM-G1, ^*bla*^TEM; lane 4: ^*bla*^CTXM-G1; Lane 5: ^*bla*^CTXM-G1, ^*bla*^TEM; Lane 6: ^*bla*^CTXM-G2, ^*bla*^CTXMG8, ^*bla*^CTXMG9; Lane 7: ^*bla*^CTXM-G1, ^*bla*^TEM; Lane 8: NCTC 13351 *E. coli* positive control for ^*bla*^TEM, NCTC 13461 *E. coli,* positive control for ^*bla*^CTX-MG1& NCTC 13368 *K. pneumonia* positive control for ^*bla*^SHV; Lane9: ATCC 25922 *E. coli* negative control; Lane 10: H_2_O negative control; M, molecular size (weight) standard marker; bp, base pairs. **b**: lanes (1–5 &7–13): ^*bla*^CTXM-G3 & ^*bla*^CTXM-G15. Lane 6: ^*bla*^CTXM-G14

Seventeen isolates were confirmed to be ESBL producers, including 13 EPEC and four EAEC. The genotypic profiles were characterized with PCR for genes encoding resistance (Fig. 2, Table 4).

**Table 4:** Distribution of *bla* genes obtained from diarrheagenic stool samples of children with acute gastroenteritis

Isolate	Gene %				
**EPEC (*n*** **= 13 ESBL)**	*CTX-M--G1(CTX-M-15, CTX-M-3)*	*TEM,CTX-M-G-1(CTX-M-15, CTX-M-3)*	*TEM, CTX-M-G-8, CTX-M-G-14,*	*TEM, SHV, CTX-M-G-1 (CTX-M-15, CTX-M-3)*	*CTX-M-G-2, CTX-M-G-8, CTX-M-G-9, CTX-M-G-14*,
	1 (7.7)	10 (76.9%)	0	1 (7.7%)	1 (7.7%)
**EAEC (*n*** **= 4 ESBL)**	3 (75%)	0	1(25%)	0	0

A combination of *bla*_CTX-MG1_ (CTX-M-15, CTX-M-3), and *bla*_TEM_ genes (76.9%) encoded the highest resistance among EPEC, followed by 7.7% of *bla*_CTX-M-G-1_ (*bla*_CTX-M-15_, *bla*_CTX-M-3_), *bla*_TEM_ and *bla*_SHV_, and 7.7% *bla*_CTX-M-G-2_, *bla*_*CTX-M-G-8*_, *bla*_*CTX-M-G-9*_ and *bla*_CTX-M-G-14_. On the other hand, the highest resistance among EAEC (75%) was encoded by to *bla*_CTX-M-G-1_ (*bla*_CTX-M-15,_*bla*_CTX-M-3_), followed by 25% of *bla*_TEM_, *bla*_CTX-M-G-8_, *bla*_CTX-M-G14_.

### Virulence genes profile

The initial diagnosis for EPEC and EAEC pathotypes was done at HMC using the *“BioFire GI Panel test”* (Biomerieux; Utah USA), which detect 22 of the most commonly pathogens associated with gastroenteritis. To better understand the diversity of the circulating strains, we further evaluated these two pathotypes by screening for the most common virulence genes as described in the literature (Table 1). EPEC strains were identified by PCR assay using primers that target the *eae* (positive; encodes intimin) and *stx* (negative) genes. We also tested EPEC strains for the presence of the *bfpA* (encoding bundlin), noting that intimin and bundlin play important roles in EPEC invasion of host cell through attachment and eternalization. On the other hand, there is no consensus on which EAEC genes are unambiguously pathogenic, and hence, we selected several genes to screen for in this study.

**Fig. 3:**
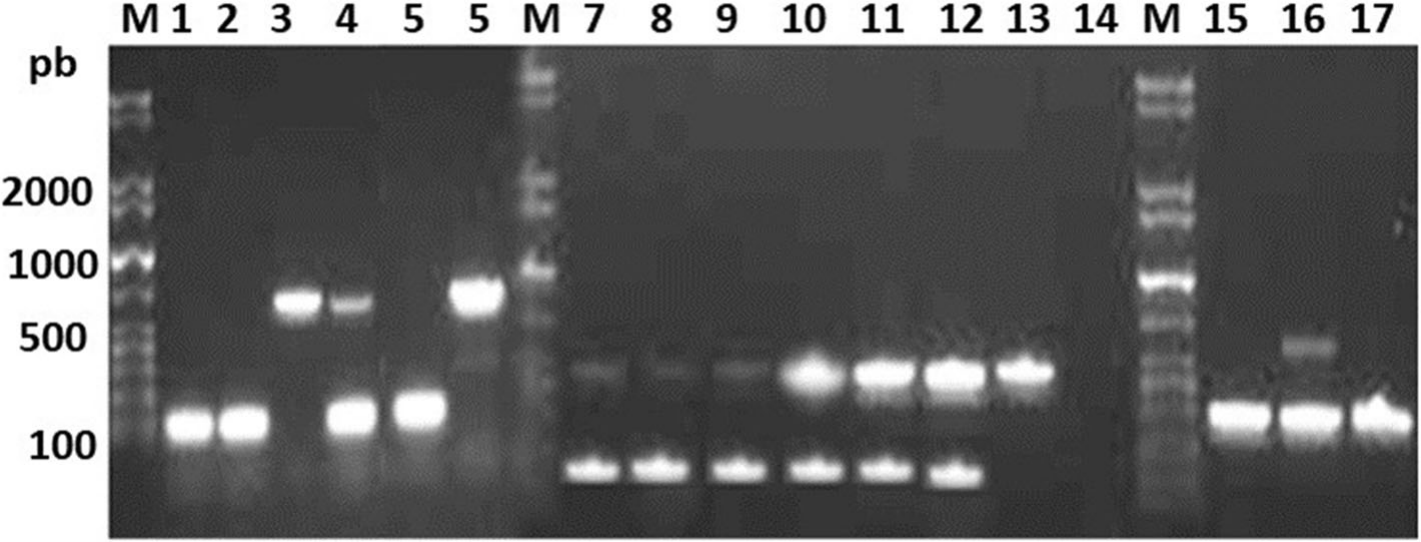
Detection of virulent genes among EAEC isolates. Representative samples are shown. Lane 1:*astA*; lane 2: *astA;* Lane 3: *aatA;* Lane 4: *astA, aatA;* Lane 5: *astA;* Lane 6: *aatA& aaiA;* Lane7: *aggR* & *east;* Lane8: *aggR* & *east;* Lane 9: *aggR* & *east;* lane 10: *aggR* & *east*; lane11: *aggR* & *east;* lane 12: *aggR* & *east;* Lane 13: *aggR;* lane 14: negative control H_2_O; Lane 15:*aap*; Lane 16: *aap* & *aatA*; Lane 17: *aap*; M, molecular size (weight) standard marker; bp, base pairs

Among the 56 isolated EPEC strains, 50 (89.3%) were positive for *eae*, and only 6 (10.7%) were positive for *eae* and *tir*. All of isolated EPEC were atypical (absence of *bfpA*). Of the 20 EAEC strains, three (15%) were positive for a combination of *astA, aap & capU*, two (10%) were positive for each of the following three combinations: 1. *aat, aai, astA, aggR, east, aap, capU;* 2. *aatA, aggR, east, aap, capU;* and 3. *aatA, aagR, east* & *aap.* No identical virulence *aap, aatA, capU* and *aggR* showed the highest frequency of 65, 60, 55 and 55% respectively. On the other hand, *east, astA*, and *aai* showed frequencies of 35, 30 and 20% respectively (Fig. 3, Table 5). The sequence analysis of the detected virulence genes found to have 99.7% similarity to (*eae*, Acc. No. MK761167); 97.4% to (*tir*, Acc.NoAF132728); 97% to (*east*, Acc. No. LC312643) 98.7% to (*capU*, Acc. No. AF134403; 95.6%); 98.6% to (*ast* ACC. No. LC312643) and 97.3% to (*aat* A, Acc. No. AY351861) sequence data were not shown.

**Table 5:** The prevalence of different virulence genes among EPEC and EAEC strains isolated from children with acute gastroenteritis

EAEC virulence genes profile	Frequency	Percentage%
*aatA, astA,aggR, aap & capU*	1	5
*aatA,astA, east, aap & capU*	1	5
*aatA, aaiA, astA, aggR & capU*	1	5
*aatA, aggR, east, aap & capU*	2	10
*aatA, astA, aggR, east, aap & capU*	1	5
*aatA, aaiA, aggR, east, aap & capU*	1	5
*aatA, aaiA, aggR, aap & capU*	1	5
*aatA, aaiA, astA, aggR, east, aap & capU*	2	10
*aatA, aggR, aap & capU*	1	5
*aatA, aggR, east & aap*	2	10
*aatA, aggR & east*	1	5
*astA, aap & capU*	3	15
*aatA, aggR &aap*	1	5
*aap & capU*	1	5
*aap*	1	5
**EPEC virulence genes**		
*eae*	50	89.3
*eae & tir*	6	10.7

## Discussion

This is one of the few studies from the MENA region that describe the AMR profile of DEC in the pediatric population and to the best of our knowledge; this is the first study that primarily investigated, the antimicrobial resistance and virulence pattern of DEC among children in Qatar. Most of the studies from the MENA region including those from Qatar [28, 29], Jeddah [13, 14], Oman [30], Kuwait [31], and Bahrain [32] focused mainly on the prevalence but not the AMR of DEC in adults and children. Very few studies from Iran [22, 33, 34], Libya [35] and Egypt [36] explored the AMR profiling among DEC. This highlights the critical need and the importance of this study, considering the multinational composition of Qatari population, where more than 80% of the population are expatriates. From this study among children suffering from AGE in Qatar, it was revealed that most of the affected group are those less than 2 years of age (59.2%), compared to older children, supporting other several studies worldwide [37–40]. Our results also depicted that EPEC was the most predominant pathogen (32%) followed by EAEC (11.43%). Accordingly, determining the virulence and AMR profile of these pathogens is very crucial in providing adequate treatment to control infections, rather than the empirical use of antibiotics that could lead to the development of resistant strains. Here, we reported a significant percentage of EPEC and EAEC isolates that are resistant to at least one antibiotic (73.7%). Overall, our results showed high resistance to first-line antibiotics such as ampicillin, tetracycline, sulfamethoxazole-trimethoprim which is consistent with old and recent reports around the globe [9, 22, 33, 36];. These first line antibiotics are widely empirically used in developing countries to treat diarrhea because of their low cost and availability [8, 41]. Alarmingly, about 40% of the isolates were MDR, with more than 20% being ESBL producers. In contrast to our findings, a relatively recent study from Tennessee, USA (Foster et al., 2015) showed that DEC isolates from children less than 12 years old with AGE were susceptible to the all antibiotics tested, with the exception of Ampicillin (5/12, 41.6%). On the other hand, several regional and international studies have reported similar findings to ours about multi-drug resistant and ESBL producers among DEC [9, 22, 42]. The worldwide prevalence of high resistance in DEC could be attributed to the inappropriate and wide use of different antibiotics to treat infection in children of a young age. Unless the patient is immunocompromised, the current practice in Qatar to treat children with AGE is primarily supportive, without the use of antibiotics. This has been the practice since the implementation of antibiotics stewardship program in Qatar’s main hospitals during 2017. Still, the acquisition of resistance could be attributed to many factors including frequent travel, the uncontrolled use of antibiotics by patients’ families that bring it without prescription from their home countries, knowing that more than 80% of Qatar population are expatriates arriving from many countries in MENA region and Southeast Asia. At the local level, our group has recently reported 27% MDR and 9% ESBL among commensal *E. coli* isolated from healthy food handlers in Qatar [43]. Accordingly, the food chain could be another factor for the dissemination of resistant *E. coli*. Particularly, the pathogenic potential of EAEC has been associated with the emergence of food-borne outbreaks, most notably in Germany in 2011 [44].

Our current findings showed that all EPEC and EAEC isolates were susceptible to meropenem, ertapenem, fosfomycin, amikacin and colistin. This might be attributed to the low prescription and consumption of these antibiotics in Qatar’s health care facilities, reflecting the compliance with the antibiotic guidelines and stewardship program. Cumulatively, our findings indicate that ampicillin and trimethoprim-sulfamethoxazole are redundant as first line empirical antibiotics for the treatment of diarrhea in acute cases and alternatives should be considered. The high prevalence (22. 4%) of ESBLs in our study carries tremendous clinical significance in terms of infection management and control. The ESBLs are primarily plasmid encoded and frequently carry genes encoding resistance to other drug classes for example, aminoglycosides [45]. Therefore, antibiotic options in the treatment of ESBL-producing organisms are extremely limited. Carbapenems are the treatment of choice for serious infections with ESBL-producing organisms, to which, DEC remains largely susceptible in this study. In the future, selective pressure on carbapenems could accelerate the development of carbapenemase resistant that already detected in several parts of the world [46–49].

Molecular analysis of resistant isolates to third generation cehalosporins indicated the presence of at least two genes that encode resistance. We observed a positive correlation between phenotypic and genotypic profiles (CTX-M, SHV and TEM) in all the isolates. In both bacterial species, EPEC and EAEC, bla_CTX-M_ was most prevalent ESBL encoding gene, which is very similar to what we have recently reported in enterobacteriaceae isolates from children suffering from urinary tract infection (23) indicating that there might be a transfer of bacteria from gut to the urinary system. We have not observed any specific pattern or trend of higher MICs and the gene detected. Presence of any of these genes does not predict the association of resistance to quinolone and aminoglycoside. Without any exception, all isolates harbored *bla*_CTX-M_ gene (100%), primarily *bla*_CTX-MG1_, which includes *bla*_CTX-M-3 &15_ (88.23%). This is in accordance with our previous study on *E. coli* ESBL producers isolated from children with urinary tract infection, where CTX-MG1 was present in more than 89% isolates [24]. That study did not investigate the type of *E. coli* causing the diseases and hence, we could not associate the present findings with our previous urinary tract study. However, it has been shown in several occasions that EAEC can be associated with urinary tract infection [50, 51].

On the other hand, this study revealed a high degree of variability of virulence markers among EAEC isolates (Fig. 3, Table 5), with 15 patterns were documented, indicating the diversity of their origin and heterogeneity with respect to virulent genes. An earlier study from Iran [33] reported similar findings about high frequency of *aggR, aap* and *astA* virulence genes from children with diarrhea. In our study, 14 isolates (70%) harbored *aggR,* indicating typical EAEC. Nine of EAEC (45%) in this study harbored *astA* gene, which was considered in the past a characteristic of EAEC strains [52]. However, this gene has been detected in only a subgroup of EAEC and has an extensive distribution among other pathogenic and non-pathogenic *E. coli* strains [53]. All 56 EPEC isolates were atypical, harboring only *eaeA* (absence of *bfpA* gene), whereas in typical EPEC both genes *eaeA* and *bfpA* are present [22, 54]. Similar to our findings, in the MENA region, atypical EPEC strains have been the most manifested in Iran (100%), Iraq (66.7%) and Kuwait (95.6%) [34, 55, 56]. They have been also reported in other countries worldwide, such as Brazil, North-West Italy, Melbourne, India [56–60]. In agreement with our findings, the atypical EPEC organisms that possess *eae* alone have been reported to be more prevalent in both developing and developed countries. Animals can be reservoirs of atypical EPEC, in contrast to typical EPEC, in which humans are the sole reservoir [61], indicating that food chain and animal contact could be other factors for the dissemination of resistant DEC among pediatrics in Qatar. In contrast with our findings, studies from Iraq and India documented atypical EPEC harboring *bfpA* without *eaeA* gene [34, 56]. Early studies conducted in England and Peru have shown that atypical EPEC is often found in children with and without diarrhea, and the pathogenic potential of atypical EPEC strains has been speculative in the past [62, 63]. A recent publication by the Global Enteric Multicenter Study (GEMS) confirmed atypical EPEC as the fifth most frequently detected pathogen in patients aged 0–11 months with AGE [20]. Further surveillance studies in Qatar that include healthy controls may provide indications on host risk factors as well as EPEC virulence factors that are associated with the disease.

This is the first study to characterize the AMR profile of DEC strains, particularly EAEC and EPEC, in Qatar. This study was restricted to only two types of DEC in children suffering from AGE, while a comprehensive study that investigate the prevalence and characteristics of more DEC types in different age groups, in healthy and diseased population, might be needed.

In conclusion, our data indicate the importance for routine laboratory detection of DEC strains coupled with performing sensitivity testing, since diagnostic tools to differentiate these *E. coli* pathotypes are not routinely readily available in all clinical laboratories in Qatar. Findings from our study could be used to develop recommendations for treating infections with DEC bacteria, especially in the pediatric populations. Knowledge of antimicrobial resistance of DEC is important in selecting the appropriate therapy in serious diarrheagenic infections and formulating local antimicrobial guidelines.

## Supplementary information

**Additional file 1.** Minimum Inhibitory Concentration range for 18 antibiotics and interpretation of the results.

## Data Availability

The datasets used and/or analyzed during the current study are available from the corresponding author on reasonable request.

## Abbreviations

AGE: Acute gastroenteritis
DDST: Double disc synergy test
DEC: Diarrheagenic *E. coli*
EAEC: Enteroaggregative *E. coli*
EHEC: Enterohemorrhagic *E. coli*
EIEC: Enteroinvasive *E. coli*
EPEC: Enteropathogenic *E. coli*
ETEC: Enterotoxigenic *E. coli*
HMC: Hamad medical corporation
MPCR: Multiplex PCR
PEC: Pediatric emergency center
SPSS: Statistical package for the social science
