# Supplementary material for: Antibiotic resistance and virulence patterns of pathogenic Escherichia coli strains associated with acute gastroenteritis among children in Qatar
Source: BMC Microbiol. 2020 Mar 6;20:54. doi: 10.1186/s12866-020-01732-8 (PMC7060563; doi:10.1186/s12866-020-01732-8)
Supplement: Supplementary file 1 — Additional file 1. Minimum Inhibitory Concentration range for 18 antibiotics and interpretation of the results. [file 12866_2020_1732_MOESM1_ESM.docx]

**Supplementary 1: Minimum Inhibitory Concentration range for 18 antibiotics and interpretation of the results.**

| **Antibiotic** | **Abbreviation** | **MIC range tested (µg/mL)** | **MIC Interpretive Standard (µg/mL)** | | |
| --- | --- | --- | --- | --- | --- |
|  |  |  | **S I R** | | |
| **Penicillin & Penicillin β-lactamase inhibitor combinations**  Ampicillin  Amoxicillin/Clavulanic acid  Piperacillin/Tazobactam | AM  AMC  TZP | 0.016-256  0.016-256  0.016-256 | ≤8  ≤8/4  ≤6/4 | 16  16/8  32/4-64/4 | ≥32  ≥32/16  ≥128/4 |
| **Aminoglycosides**  Amikacin | AK | 0.016-256 | ≤16 | 32 | ≥64 |
| **Quinolone**  Ciprofloxacin | CIP | 0.002-32 | ≤1 | 2 | ≥4 |
| **Folate pathway inhibitors**  Trimethoprim/Sulfamethoxazole | SXT | 0.002-32 | ≤2/38 | - | ≥4 |
| **Cephalosporin**  Cephalothin  Cefuroxime  Ceftriaxon  Cefepime | KF  CXM  TX  FEP | 0.016-256  0.016-256  0.016-256  0.016-256 | ≤8  ≤8  ≤1  ≤2 | 16  16  2  - | ≥32  ≥32  ≥4  ≥16 |
| **Chloramphenicol** |  | 0.016-256 | ≤8 | 16 | ≥32 |
| **Polymyxin**  Colistin* | CS | 0.016-256 | ≤2 | - | >2 |
| Fosfomycin | FOS | 0.064-1024 | ≤64 | 128 | ≥256 |
| **Glycylcycline**  Tigecycline*  Tetracycline | TGC  TC | 0.016-256  0.016-256 | ≤1  ≤4 | 2  8 | >2  ≥16 |
| Nitrofurantoin | F | 0.032-512 | ≤32 | 64 | ≥128 |
| **Carbapenems**  Ertapenem  Meropenem | ETP  MRP | 0.002-32  0.002-32 | ≤0.5  ≤1 | 1  2 | ≥2  ≥4 |

*No CLSI interpretive criteria are available; therefore, provisional breakpoints by the European committee on Antimicrobial Susceptibility Testing (EUCAST 2017) breakpoint tables were consulted.
